# Plasma Leucine-Rich α-2-Glycoprotein 1 Predicts Cardiovascular Disease Risk in End-Stage Renal Disease

**DOI:** 10.1038/s41598-020-62989-7

**Published:** 2020-04-06

**Authors:** Feng-Jung Yang, Chun-Yih Hsieh, Kai-Hsiang Shu, I-Yu Chen, Szu-Yu Pan, Yi-Fang Chuang, Yen-Ling Chiu, Wei-Shiung Yang

**Affiliations:** 1 Graduate Institute of Clinical Medicine, College of Medicine, National Taiwan University, Taipei, Taiwan; 2 Department of Internal Medicine, National Taiwan University Hospital Yun Lin Branch, Douliu, Taiwan; 3 Genome and Systems Biology Degree Program, Academia Sinica and National Taiwan University, Taipei, Taiwan; 4 Division of Nephrology, Department of Internal Medicine, Linkou Chang Gung Memorial Hospital, Taoyuan, Taiwan; 5 Department of Internal Medicine, Far Eastern Memorial Hospital, New Taipei, Taiwan; 6 Graduate Institute of Immunology, College of Medicine, National Taiwan University, Taipei, Taiwan; 7 Department of Epidemiology, School of Public Health, National Yang Ming University, Taipei, Taiwan; 8 Graduate Program in Biomedical Informatics, College of Informatics, Yuan Ze University, Taoyuan, Taiwan; 9 Department of Internal Medicine, National Taiwan University Hospital, Taipei, Taiwan

**Keywords:** Predictive markers, Haemodialysis

## Abstract

Plasma leucine-Rich α-2-glycoprotein 1 (LRG1) is an innovative biomarker for inflammation and angiogenesis. Many adverse pathophysiological changes including inflammation, atherosclerosis, and premature mortality is associated with End-stage renal disease (ESRD). However, whether levels of plasma LRG1 correlate with the co-morbidities of ESRD patients is unknown. Plasma LRG1 and high-sensitivity C-reactive protein (hsCRP) were analyzed by ELISA in 169 hemodialysis patients from the Immunity in ESRD (iESRD) study. Patient demographics and comorbidities at the time of enrollment were recorded. Peripheral blood monocyte and T cell subsets were assessed by multicolor flow cytometry. In the univariate analysis, a higher level of LRG1 was associated with the presence of cardiovascular disease (CVD) and peripheral arterial occlusive disease (PAOD). In multivariate logistic regression models, higher LRG1 tertile was significantly associated with PAOD (odds ratio = 3.49) and CVD (odds ratio = 1.65), but not with coronary artery disease, history of myocardial infarction, or stroke after adjusting for gender, diabetes, hemoglobin, albumin, calcium-phosphate product, and level of hsCRP. In addition, the level of LRG1 had a positive correlation with IL-6, hsCRP, and also more advanced T cell differentiation. The association suggests that LRG1 participates in the progression of atherosclerosis by inducing inflammation. Therefore, the role of LRG1 in coexisting inflammatory response should be further investigated in the pathogenesis of cardiovascular morbidity and mortality in patients with ESRD.

## Introduction

Cardiovascular mortality in patients with end-stage renal disease (ESRD) remains the leading cause of death^1,2^. These patients exhibit a strikingly higher risk (20–400 folds) of cardiovascular mortality compared to age-matched health subjects without any kidney disease^3^. Traditional risk factors as well as non-traditional risk factors, such as inflammation, are believed to contribute to the excessively heavy burden of cardiovascular disease in chronic kidney disease (CKD) and ESRD patients^3^. The pro-inflammatory microenvironment is collectively caused by uremic milieu, infection, and tissue ischemia even before the initiation of dialysis^4^.

Innate immunity is the evolutionarily conserved host response mediated by pattern recognition receptors (PRRs), which bind endogenous or exogenous ligands and initiate downstream signaling pathways to establish an immediate responses as the first line of defense. Two types of PRRs, Toll-like receptors (TLRs) over the cell surface and inflammasomes in the cytoplasm can interact with endogenous ligands. They are involved in the development of pro-inflammatory microenvironment in renal failure. Activation of these innate immune pathways is associated with hypertension, development of atherosclerotic plaques, vascular calcification and clinically significant cardiovascular diseases in CKD patients. However, detailed molecular mechanisms underlying the contribution of the innate immune system to CKD-associated CVDs are still poorly understood^5^.

The leucine-rich repeats (LRRs)-containing domain is evolutionarily conserved in PRRs and are responsible for ligand binding^6^. First identified in 1977, leucine-rich α-2-glycoprotein-1 (LRG1 or LRG) is a member of the LRR family of proteins that are composed of repeating 20–30 amino acid stretches that are rich in leucine- a kind of hydrophobic amino acid^7^. This 50 kDa glycosylated protein has 347 amino acids and the serum concentration in healthy individuals ranges from 10–50 µg/mL^8,9^. Expression of LRG1 in the liver is upregulated by lipopolysaccharide as well as mediators of the acute-phase response such as interleukin-6 (IL-6)^10^. LRG1 may serve as an acute-phase reactant, and has been proposed as a biomarker of inflammatory diseases such as systemic lupus erythematosus, rheumatoid arthritis, asthma, and ulcerative colitis^10–12^. In addition, LRG1 may play a role in pathogenic angiogenesis and is associated with vascular endothelial dysfunction, arterial stiffness, and peripheral arterial occlusive disease in patients with type 2 diabetes^13^. In the kidney, LRG1 protein expression is upregulated in injured proximal tubules, distal tubules, and collecting ducts in albumin-overloaded mice^14^. Glorieux et al. performed high-resolution LC-MS/MS analysis of the plasma proteomes in patients with moderate to advanced CKD. The level of LRG1 protein was two-times higher in ESRD patients treated with hemodialysis than in CKD stage 2/3 patients. ELISA measurement performed in the validation cohort revealed an inverse correlation between plasma LRG1 level and eGFR. Moreover, a higher LRG1 level was independently associated with an increased risk of all-cause mortality in hemodialysis patients^15^.

To our knowledge, the relationship between LRG1 and cardiovascular events in patients with ESRD remains unclear. Here, we investigate the association between plasma LRG1 with cardiovascular comorbidities in hemodialysis patients of iESRD cohort.

## Methods

### Participants

This analysis is based on the subjects enrolled in the Immunity in ESRD (iESRD) cohort that were recruited from the National Taiwan University Hospital Yun Lin branch^16^. All subjects were screened for eligibility and signed informed consent forms to join this study. The patients who were recently hospitalized (within three months), had acute or chronic infections receiving antibiotic treatment, incomplete blood test results, suboptimal sample quality or quantity were excluded. There were 169 eligible adult ESRD subjects who received regular hemodialysis thrice weekly for at least one year. The patients were cared by their primary care nephrologists according to the Kidney Disease Outcomes Quality Initiative (KDOQI) guidelines. The study is approved by the Institutional Review Board of National Taiwan University Hospital (NTUYL 201511092 RINA). All research procedures followed the directives of the Declaration of Helsinki.

### Data collection and laboratory exams

We collected peripheral blood samples at the beginning of hemodialysis during the first session of the week. Hemoglobin level, white blood cell counts, blood biochemistry including blood urea nitrogen (BUN), creatinine, albumin, total cholesterol, triglyceride, calcium, and phosphate were measured. Kt/V and normalized protein catabolic rate (nPCR) were calculated to represent dialysis adequacy and dietary protein intake; high sensitivity C-reactive protein (hsCRP) nephelometry (from Siemens) and intact-parathyroid hormone (i-PTH) immunoradiometric assay (from CISBio International) were assessed at the same time. Peripheral blood mononuclear cells (PBMCs) were isolated and submitted for flow cytometry analysis. LRG1 was measured by ELISA (from RayBio Human assay). The plasma levels of inflammatory cytokines, TNF-α and IL-6, were assayed with human TNF-α Quantikine ELISA kit and human IL-6 Quantikine HS ELISA kit and, respectively (from R&D systems).

### Immunophenotyping and multicolor flow cytometry

The immunophenotyping methods and gating strategy were described in the previous iESRD study, which was published recently with more detail and included the representative figure depicting gating strategies^16^. In brief, we obtained peripheral blood mononuclear cell (PBMC) by using Ficoll-Paque gradient centrifugation following the manufacturer’s protocol (from GE Healthcare) and send into flow cytometry to identify distinct cell types based on surface marker. The antibodies were presented with the name (flow channel, clone). After gated on singlets, we used CD3(AF700, clone UCHT1) to identify CD3 + cells from the lymphocytes gate. Afterwards, we determined CD4 + and CD8 + T cells by CD4(Percpcy5.5, clone OKT4) and CD8(APC-Cy7, clone SK1) expression and T cell differentiation state by CCR7(APC, clone G043H7) and CD45RA(Alexa488, clone HI100) with CD28(Pe-Cy7, clone 28.2). The expression patterns of specific T cell subsets in progressive differentiation status are as the following: CD45RA + CCR7 + as Naïve T cells (T_naive_); CD45RA-CCR7 + as central memory T cells (T_CM_); CD45RA-CCR7- as effector memory T cell (T_EM_); and CD45RA + CCR7- as terminal effector T cells with CD45RA re-expression(T_EMRA_).

We performed Monocyte staining with a pan-monocyte marker CD86 to identify total monocytes^17^. After gating on the appropriate forward scatter/side scatter, we identified monocytes by expression of CD86(PE, clone IT2.2), and classified three types: CD14 + + CD16- as classical monocytes, CD14 + + CD16 + as intermediate monocytes, and CD14 + CD16 + + as non-classical monocytes by CD14(FITC, clone M5E2) and CD16(APC, clone 3G8) co-expression patterns. In general, the immune cell percentage calculated for a specific immune cell subset refers to the percentage of cells among the mother population, as assessed by flow cytometry. Data acquisition was performed using a Beckman Coulter MoFlo^TM^-XDP multicolor flow cytometer located at the Far Eastern Memorial Hospital.

### Cardiovascular co-morbidities

Baseline demographics and clinical characteristics including diabetic status and history of myocardial infarction (MI) were retrieved by thorough review of medical records. The presence of coronary artery disease (CAD) was defined as either 1) documented perfusion defect on stressed cardiac nuclear scan or 2) > 50% stenosis of at least one coronary artery on angiography. Peripheral arterial occlusive disease (PAOD) was defined as 1) previous non-traumatic leg amputation, surgical or endovascular revascularization of lower limbs or 2) ankle brachial pressure index (ABPI) of ≤ 0.90 in one or both legs with presence of intermittent claudication or 3) previous carotid endarterectomy. The diagnosis of congestive heart failure (CHF) was made on clinical ground according to symptoms and signs resulting from an abnormal cardiac structure or function. Stroke includes ischemic and hemorrhagic events. Cardiovascular disease (CVD) was defined as having documented CHF, CAD, PAOD, or stroke.

### Statistical analyses

Baseline characteristics of our cohort were described as frequency for categorical variables and mean ± standard deviation for continuous variables. These variables were analyzed by Chi-square test and ANOVA, respectively. Evaluating the correlation of LRG1 level with inflammatory markers, frequencies and absolute numbers of immune cell subsets, Pearson correlation was applied.

Logistic regression models were used to calculate the predictive value of LRG1 levels on cardiovascular comorbidities. Univariate logistic regression was performed to calculate the p for trend value to determine the relationship between LRG1 tertiles and individual comorbidity. Multivariate logistic regression analysis with two models: model 1 (age, gender, diabetes mellitus) and model 2 (age, gender, diabetes mellitus, albumin, hemoglobin, calcium- phosphate product and hsCRP), were used to investigate the association between LRG1 tertiles or concentration with cardiovascular comorbidities.

All statistical tests were performed by two-tailed, and a p value of less than 0.05 was considered to be significant. Statistical analyses were performed with SPSS Statistics Version 26 (IBM) and Prism Version 8.3.1(322) (GraphPad).

## Results

### Patient demographics and clinical characteristics

There were 169 adult ESRD patients treated with chronic hemodialysis enrolled in this study. Their age was 62.6 ± 12.2 years and 42% of patients were male. Their dialysis vintage was 5.3 years in average and 41% had diabetes. The overall frequency of cardiovascular disease in this cohort was 27%; coronary artery disease (CAD) and congestive heart failure (CHF) were the most prevalent, followed by peripheral arterial occlusive disease (PAOD), history of myocardial infarction (MI), and stroke.

### Plasma LRG1 levels in ESRD patients

The mean plasma LRG1 levels in our ESRD patients was 67.73 ± 15.10 µg/mL. We further stratified patients into three groups according to LRG1 tertiles and investigated the differences of patient characteristics and blood biochemistry. The patients in the highest (3^rd^) LRG1 tertile had significantly lower levels of hemoglobin, serum albumin, and creatinine, while there was no difference regarding age, gender, markers of bone-mineral disorder, Kt/V, and nPCR among three groups. The 3^rd^ tertile group also had higher hsCRP and IL-6 levels than the other two groups (Table 1).

**Table 1:** Baseline demographic, clinical and laboratory parameters stratified by plasma LRG1 level in ESRD patients.

	Total	1^st^ Tertile (n = 57)	2^nd^ Tertile (n = 56)	3^rd^ Tertile (n = 56)	p value
LRG1 (μg/mL)	67.7 (15.1)	52.3 (8.0)	67.8 (3.3)	83.4 (11.0)	<0.001*
Age (years)	62.6 (12.2)	61.1 (12.2)	61.6 (12.5)	65.2 (11.5)	0.159
Dialysis vintage (years)	5.3 (4.0)	5.3 (3.8)	5.7 (4.8)	4.9 (3.5)	0.593
Male (%)	42	40	39	46	0.714
Diabetes (%)	41	32	39	54	0.055
WBC (x 1000/μL)	6.3 (2.0)	6.0 (1.8)	6.3 (2.0)	6.7 (2.2)	0.193
Hemoglobin (g/dL)	10.4 (1.2)	10.7 (0.9)	10.5 (1.2)	10 (1.4)	0.013*
Albumin (g/dL)	3.91 (0.42)	3.99 (0.36)	4.00 (0.36)	3.75 (0.50)	0.001*
BUN (mg/dL)	84.3 (19.5)	83.4 (14.5)	89.1 (19.1)	80.5 (23.4)	0.061
Creatinine (mg/dL)	11.7 (2.6)	11.9 (2.4)	12.3 (2.6)	10.8 (2.8)	0.011*
Kt/V (Gotch)	1.46 (0.18)	1.50 (0.18)	1.47 (0.18)	1.42 (0.17)	0.058
nPCR (g/Kg)	1.22 (0.64)	1.29 (0.33)	1.33 (0.38)	1.03 (0.97)	0.099
Total cholesterol (mg/dL)	141.9 (31.5)	140.1(29.9)	142.6 (32.7)	143.0 (32.4)	0.870
Triglyceride (mg/dL)	130.5 (78.1)	130.9 (86.3)	134.3 (71.3)	126.0 (76.8)	0.860
Calcium (mg/dL)	9.6 (0.8)	9.8 (0.9)	9.6 (0.7)	9.4 (0.7)	0.100
Phosphate (mg/dL)	4.9 (1.5)	4.9 (1.6)	5.2 (1.3)	4.6 (1.6)	0.122
Intact PTH (pg/mL)	355.8 (452.1)	290.5 (326.9)	381.0 (487.3)	399.5 (524.6)	0.403
hsCRP (mg/dL)	0.81 (1.46)	0.31 (0.34)	0.53 (0.86)	1.59 (2.16)	<0.0001
IL-6 (pg/mL)	4.79 (3.65)	3.54 (1.61)	3.56 (2.62)	6.62 (4.60)	<0.0001
TNF-α (pg/mL)	6.29 (1.85)	6.43 (1.52)	6.17 (1.93)	6.27 (2.06)	0.88
CAD (%)	22	14	25	27	0.209
CHF (%)	21	19	21	21	0.950
MI (%)	5	2	7	7	0.341
PAOD (%)	7	0	7	13	0.025*
Stroke (%)	4	4	4	5	0.858
CVD (%)	27	16	29	37	0.033*

Demographic and clinic data were compared across among LRG1 tertiles in 169 ESRD patients in the iESRD cohort. Continuous values are expressed as means (SD). nPCR: normalized protein catabolic rate; CAD: coronary artery disease; CHF: congestive heart failure; MI: myocardial infarction; PAOD: peripheral artery occlusive disease; CVD: cardiovascular disease. *p value < 0.05.

### Elevated plasma LRG1 is associated with systemic inflammation in ESRD

**Figure 1:**
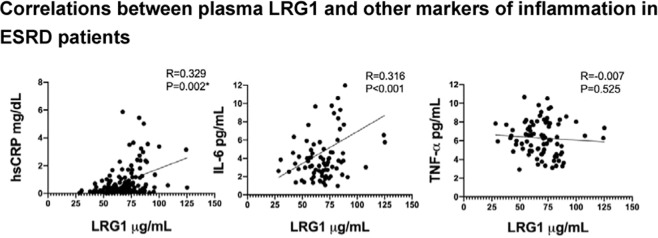
Correlations between plasma LRG1 and other markers of inflammation in ESRD patients. Pearson correlation was applied to investigate the relationship between plasma LRG1 level and inflammatory markers including hsCRP (mg/dL), IL-6 (pg/mL) and TNFα (pg/mL).

We examined the association between plasma LRG1 and circulating inflammatory markers. As shown in Fig. 1, there is a significant association between plasma LRG-1 with IL-6 (r = 0.329, p = 0.002*) as well as with hsCRP (r = 0.316, p < 0.001). In addition, the level of hsCRP was strongly correlated with IL-6 (r = 0.698, p < 0.001). On the other hand, no such relationship was observed between plasma LRG1 and TNF-α (p > 0.05). Elevated hsCRP and IL-6 levels in chronically stable hemodialysis patients signify the presence of chronic inflammation. Therefore, the coexistence of LRG1 increase level may also reflect the degree of inflammation in ESRD patients.

### Higher LRG1 is associated with lower central memory T cells in ESRD

Accumulating evidence revealed that certain T cell and monocyte subsets with more advanced differentiation are linked with cardiovascular disease and/or atherosclerosis^18–20^. Central memory T cells (T_CM_) constitutively express CCR7 and CD62L, which are required for cell extravasation into the secondary lymphoid system before migration to T cell areas^21^. The T_CM_ is rapidly transformed into T_EM_ without antigenic stimulation, and further into T_EMRA_ cells. T_EM_ and T_EMRA_ cells remain CCR7- and maintain both memory and flexibility of cytokine gene expression, but they also upregulate CX3CR1 which allows them to bind to vascular endothelium and promote atherosclerosis. Many previous studies, including ours, indicated that T cells with advanced differentiation status accumulate in ESRD patients and the phenomenon is independently associated with cardiovascular diseases^16,20,22^.

To understand the relationship between LRG1 and T cell differentiation, we analyzed the immunophenotyping results of peripheral blood T cell and monocyte in 169 ESRD patients. On the other hand, we found the relationship between plasma LRG1 and each immune cell subset, in either relative (cell percentage of mother population) or absolute (cell number per µL of blood) terms. The T cells were divided into four subsets: CCR7 + CD45RA + as T_NAIVE_, CCR7 + CD45RA- as T_CM_, CCR7-CD45RA- as T_EM_, and CCR7-CD45RA as T_EMRA_ subsets. As shown in Table 2, the level of LRG1 is associated with alteration of T cell immunophenotyping in ESRD. Patients with lower T_CM_ percentages of CD4 + and CD8 + T cells and, lower T_NAIVE_ percentages of CD8 + T cells exhibit higher titers of plasma LRG1. Lower absolute T_CM_ cell counts of CD4 + and CD8 + T cell and lower absolute T_NAIVE_ cell counts of CD8 + T cell had similar trends (Table 2). In contrast, monocyte subsets in our cohort did not correlate with the plasma LRG1 level. As far as we know, this is the first report demonstrating the positive relationship between the level of plasma LRG1 and enhanced differentiation status of peripheral blood CD4 + and CD8 + T cells.

**Table 2:** Correlations between plasma LRG1 concentration and immune cell levels in ESRD patients.

Cell type	Cell frequency		Cell type	Absolute cell number	
	R	p value		R	p value
CD4 + T cells			CD4 + T cells		
T_NAIVE_	−0.102	NS	T_NAIVE_	0.004	NS
T_CM_	−0.22	0.004*	T_CM_	−0.211	0.006*
T_EM_	0.007	NS	T_EM_	0.049	NS
T_EMRA_	0.069	NS	T_EMRA_	0.01	NS
CD8 + T cells			CD8 + T cells		
T_NAIVE_	−0.179	0.020*	T_NAIVE_	−0.187	0.015*
T_CM_	−0.173	0.039*	T_CM_	−0.15	0.001**
T_EM_	0.084	NS	T_EM_	−0.051	NS
T_EMRA_	0.05	NS	T_EMRA_	0.041	NS
Monocytes			Monocytes		
Classical	0.008	NS	Classical	0.068	NS
Intermediate	0.036	NS	Intermediate	0.076	NS
Non-Classical	-0.019	NS	Non-Classical	-0.083	NS

Pearson correlation was applied to investigate the relationship between the concentration of LRG1 and immune cell levels (CD4 + , CD8 + T cell and monocytes), including the percentages as well as absolute cell counts of naïve (TNAIVE), central memory (TCM), effector memory (TEM), terminally differentiated (TEMRA) subsets, and three monocyte subsets (classical monocytes, intermediate monocytes, non-classical monocytes). *p value <0.05. **p value <0.001. NS: non-significant, p value > 0.1.

### The frequency of cardiovascular comorbidities stratified by plasma LRG1 levels

**Figure 2:**
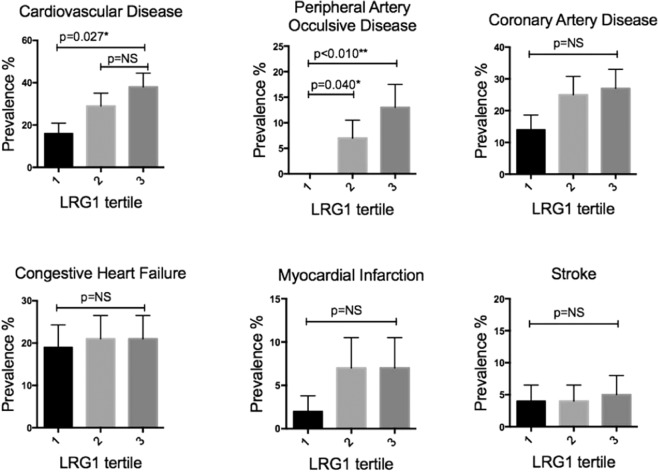
Prevalence of cardiovascular comorbidities stratified by plasma LRG1 level in ESRD patients. Percentage and standard error of patients with each specified co-morbidity among each plasma LRG1 tertile group is shown. Comparisons were performed by chi-square test. *p value <0.05. NS: non-significant, p value > 0.05.

We next compared the prevalence of cardiovascular complications among patients with different plasma LRG1 tertiles. As shown in Table 1 and Fig. 2, patients within the highest plasma LRG1 tertile had higher rates of PAOD and CVD. However, the prevalence of CAD, MI, and stroke were not different among three groups. Interestingly, when the cohort was stratified by hsCRP level instead of LRG1, a similar trend for PAOD, in that, higher hsCRP tends to be associated with more co-morbidities especially PAOD (Supplementary Fig. 1). However, when patients in the highest tertile of hsCRP level did not have higher percentage of co-morbidities as demonstrated by LRG1. When each comorbidity was analyzed in univariate regression models with LRG1 tertile. We found higher LRG1 tertile to be significantly associated with PAOD (p for trend = 0.007*, odds ratio = 3.49), CVD (p for trend = 0.010*, odds ratio = 1.65), CAD (p for trend = 0.010*, odds ratio = 1.34,), but not with history of MI or stroke.

### Plasma LRG1 level is independently associated with PAOD and prevalent CVD

Finally, we tested the independent association of plasma LRG1 with PAOD and CVD. As shown in Table 3, higher LRG1 tertile independently associated with both PAOD and CVD in two models of multivariate logistic regression analysis after adjusted with age, gender, DM with/without the other risk factors including albumin, hemoglobin, calcium phosphate product and hsCRP. Similar trends were found with absolute LRG1 level when used into the same models. Higher LRG1 level independently associated with both PAOD and CVD (Table 4).

**Table 3:** Associations between plasma LRG1 tertile with peripheral arterial occlusive disease (PAOD) and cardiovascular disease (CVD).

*Independent variables in model (dependent variable: PAOD)*	Model 1		Model 2	
	*Odds Ratio (95% CI)*	*p value*	*Odds Ratio (95% CI)*	*p value*
Age	1.10 (1.02–1.18)	0.012*	1.14 (1.03–1.25)	0.01*
Gender (Male)	1.79 (0.43–7.42)	0.425	1.96 (0.46–8.32)	0.362
Diabetes	4.29 (0.89–20.80)	0.07	4.36 (0.80–23.70)	0.088
Albumin (g/dL)			1.34 (0.20–9.08)	0.766
Hemoglobin (g/dL)			2.28 (1.07–4.83)	0.033*
Ca×P product (mg^2^/dL^2^)			1.28 (0.88–1.86)	0.2
hsCRP (mg/dL)			0.99 (0.93–1.05)	0.708
Plasma LRG1 tertile	2.97 (1.07–8.25)	0.036*	3.49 (1.13–10.76)	0.03*
***Independent variables in model (dependent variable: CVD)***	***Odds Ratio (95% CI)***	***p value***	***OR (95% CI)***	***p value***
Age	1.04 (1.01–1.07)	0.016*	1.05 (1.01–1.09)	0.011*
Gender (Male)	0.72 (0.33–1.55)	0.395	0.70 (0.32–1.53)	0.371
Diabetes	2.38 (1.13–5.02)	0.023*	2.41 (1.12–5.15)	0.024*
Albumin (g/dL)			1.57 (0.54–4.58)	0.412
Hemoglobin (g/dL)			1.01 (0.73–1.41)	0.951
Ca×P product (mg^2^/dL^2^)			1.02 (0.78–1.33)	0.883
hsCRP (mg/dL)			1.01 (0.98–1.03)	0.736
Plasma LRG1 tertile	1.57 (1.00–2.47)	0.049*	1.65 (1.02–2.68)	0.043*

Multivariate logistic regression analysis with two models: model 1 (age, gender, diabetes mellitus) and model 2 (age, gender, diabetes mellitus, albumin, hemoglobin, calcium phosphate product and hsCRP), were used to investigate the association between LRG-1 tertiles and baseline co-morbidities.

**Table 4:** Associations between plasma LRG1 concentration with peripheral arterial occlusive disease (PAOD) and cardiovascular disease (CVD).

*Independent variables (dependent variable: PAOD)*	Model 1		Model 2	
	*Odds Ratio (95% CI)*	*p value*	*Odds Ratio (95% CI)*	*p value*
Age	1.11 (1.03–1.20)	0.009*	1.14 (1.04–1.26)	0.009*
Gender (Male)	1.76 (0.42–7.39)	0.439	1.97 (0.46–8.50)	0.365
Diabetes	7.14 (1.28–39.89)	0.025*	7.67 (1.16–50.51)	0.034*
Albumin (g/dL)			1.17 (0.16–8.73)	0.878
Hemoglobin (g/dL)			2.15 (1.03–4.51)	0.042*
Ca×P product (mg^2^/dL^2^)			0.99 (0.94–1.06)	0.842
hsCRP (mg/dL)			1.31 (0.90–1.92)	0.164
Plasma LRG1 (μg/ml)	1.06 (1.02–1.12)	0.011*	1.07 (1.02–1.12)	0.012*
***Independent variables (dependent variable: CVD)***	***Odds Ratio (95% CI)***	***p value***	***OR (95% CI)***	***p value***
Age	1.04 (1.01–1.07)	0.017*	1.05 (1.01–1.09)	0.011*
Gender (Male)	0.70 (0.32–1.52)	0.365	0.68 (0.31–1.50)	0.341
Diabetes	2.59 (1.23–5.46)	0.013*	2.67 (1.24–5.73)	0.012*
Albumin (g/dL)			1.52 (0.52–4.49)	0.446
Hemoglobin (g/dL)			1.01 (0.72–1.41)	0.963
Ca×P product (mg^2^/dL^2^)			1.01 (0.98–1.03)	0.661
hsCRP (mg/dL)			1.02 (0.78–1.33)	0.884
Plasma LRG1 (μg/ml)	1.03 (1.00–1.05)	0.028*	1.03 (1.00–1.06)	0.025*

Multivariate logistic regression analysis with two models: model 1 (age, gender, diabetes mellitus) and model 2 (age, gender, diabetes mellitus, albumin, hemoglobin, calcium phosphate product and hsCRP), were used to investigate the association between LRG1 concentration and baseline co-morbidities.

## Discussion

Emerging evidence demonstrated that plasma LRG1 serves as a serum biomarker for various inflammatory and autoimmune diseases. LRG1 expression is upregulated at sites of inflammation, resulting from induction by various inflammatory cytokines in different cell types. In the present study, we successfully demonstrated strong association between high plasma LRG1 with systemic inflammation and increased risk for PAOD and CVD in ESRD patients from the iESRD cohort. This suggests a potential mechanistic link between LRG1, inflammation and atherosclerosis in this patient population, and broadens the role of LRG1 in diseases characterized by chronic inflammation.

An increased LRG1 level has been observed in obese and morbidly obese subjects and is associated with a higher hsCRP level^23^. While there was no correlation between LRG1 titer and weight or lipid profile in our study, LRG1 was significantly linked with systemic inflammation and enhanced T cell differentiation. Enhanced T cell differentiation, or T cell immunosenescence, is associated with the diminishment of T_NAIVE_ cells capable of vaccination response and the expansion of T_EMRA_ cells which have the potential to mediate vascular endothelium binding and promotes atherosclerosis^24^. Our recent study conducted in the whole iESRD cohort also showed that advanced T cell differentiation and T_EMRA_ cell expansion is positively associated with systemic inflammation^25^. As a result, it is plausible that systemic inflammation in ESRD drives both the increase in LRG1 and alteration in T cell differentiation.

Chronic inflammation is a hallmark of CKD and ESRD^26–28^. In ulcerative colitis, LRG1 level is elevated and parallel to disease activity. LRG1 expression can be induced by TNF-α, IL-6, and IL-22 in human colon adenocarcinoma cell line. However, in IL-6 deficient mice treated with lipopolysaccharide or induced colitis, LRG1 increase can still be observed, suggesting there may be an IL-6 independent mechanism to upregulate LRG1^10^. Recent studies showed that LRG1 promotes pathogenic neovascularization by binding to endoglin and subsequent activation of the TGF-β signaling pathway in endothelial cells^29,30^. In a 3-year prospective study of type 2 diabetes patients, a higher LRG1 level at baseline predicts higher risk for progression of albuminuria as well as eGFR decline independently^31^. LRG1 is upregulated in the glomerular endothelial cells isolated from streptozotocin-induced diabetic eNOS null mice as the model of diabetic nephropathy^32^. Moreover, LRG1 independently predicts the progression of albuminuria more strongly than traditional risk factors, including baseline eGFR and urine albumin to creatinine ratio^31^. In previous work, the level of plasma LRG1 was found to neither be affected by age nor by dialysis vintage on ESRD patients. LRG1 might be the link between the inflammation and increased cardiovascular mortality in ESRD^15^.

While LRG1 is upregulated by inflammation, the molecular mechanism of LRG1 in inflammatory disease-related pathologies may be different from inflammatory cytokines. The mechanism by which LRG1 leads to higher risk of cardiovascular morbidity and mortality still requires further investigation. In patients with different types of heart failure, high LRG1 level consistently identified patients with high brain natriuretic peptide (BNP) levels and can even identify heart failure independently from BNP^33^. In the same study, LRG1 expression in the myocardium was found to be positively associated with expression levels of transforming growth factor β receptor and α-smooth muscle actin. These findings indicate that LRG1 may actively participate in vascular remodeling during heart failure. Indeed, in mouse models of myocardial infarction, LRG1 ablation results in aggravated myocardial fibrosis and heart dysfunction after infarction^34^. Because transplantation of wild type mouse bone marrow alleviated the change, the authors concluded that LRG1 production from heart-infiltrating myeloid cells improves local angiogenesis, suppresses cardiac remodeling and protects against cardiovascular diseases. The involvement of LRG1 in angiogenesis may heighten its importance in cardiovascular disease beyond inflammation.

The prevalence of PAOD in ESRD population range from 5.5 to 23% by history and physical examination solely, and 16-48% by measuring ankle brachial index (ABI), toe-brachial index (TBI), or pulse volume recordings^35^. Although ABI value may increase in the presence of vascular calcification, an ABI of <0.9 independently predicts failure of arteriovenous access, cardiovascular well as all-cause mortality in hemodialysis patients^36–38^. Our study revealed a particularly high rate of PAOD in patients with higher plasma LRG1 levels, even after adjustment for diabetes, albumin and hsCRP levels. Higher LRG1 levels are also associated with high hsCRP and IL-6, suggesting the pivotal role of inflammation in the development of PAOD. Therefore, our study suggests LRG1 as a marker of inflammation and PAOD in ESRD patients.

The limitation of this study is the cross-sectional design. Therefore, a causal relationship between levels of plasma LRG1 and cardiovascular events could not be established. Furthermore, it remains unclear if LRG1 level will fluctuate in ESRD patients. Ongoing follow-up of patients in the iESRD cohort may provide further insights and the correlation of LRG1 with cardiovascular mortality.

In conclusion, this study, for the first time, demonstrates that an elevated plasma LRG1 level is independently associated with higher prevalence of PAOD and CVD in ESRD patients treated with hemodialysis. The level of plasma LRG1 is also significantly correlated with inflammation, represented by IL-6 and hsCRP levels, as well as T cell immunosenescence, which collectively contributes to the development of cardiovascular pathology. LRG1 may serve as a predictive factor for cardiovascular disease in ESRD patients and its pathogenic role warrants further investigation.

## Declarations

### Ethics approval and consent to participate

The study is approved by NTUH’s institutional ethical committee (NTUYL 201511092 RINA) and informed consent was acquired from all participants.

## Supplementary information

Supplementary Figure 1.

## Data Availability

Original flow cytometry data will be available upon request.
