# Supplementary material for: Plasma Leucine-Rich α-2-Glycoprotein 1 Predicts Cardiovascular Disease Risk in End-Stage Renal Disease
Source: Sci Rep. 2020 Apr 6;10:5988. doi: 10.1038/s41598-020-62989-7 (PMC7136266; doi:10.1038/s41598-020-62989-7)
Supplement: Supplementary file 1 — Supplementary Figure 1. [file 41598_2020_62989_MOESM1_ESM.docx]

**Plasma Leucine-Rich α-2-Glycoprotein 1 Predicts Cardiovascular Disease Risk in End-Stage Renal Disease**

Feng-Jung Yang^1,2^, Chun-Yih Hsieh^3,4^, Kai-Hsiang Shu^5,6^, I-Yu Chen^5^, Szu-Yu Pan^5^, Yi-Fang Chuang^7^, Yen-Ling Chiu^1,5,8*^, Wei-Shiung Yang ^1,3,9*^

^1^Graduate Institute of Clinical Medicine, College of Medicine, National Taiwan University, Taipei, Taiwan

^2^Department of Internal Medicine, National Taiwan University Hospital Yun Lin Branch, Douliu, Taiwan

^3^Genome and Systems Biology Degree Program, National Taiwan University

^4^Division of Nephrology, Department of Internal Medicine, Linkou Chang Gung Memorial Hospital, Taoyuan, Taiwan

^5^Department of Internal Medicine, Far Eastern Memorial Hospital, New Taipei City, Taiwan

^6^Graduate Institute of Immunology, College of Medicine, National Taiwan University, Taipei, Taiwan

^7^Department of Epidemiology, National Yang Ming University School of Public Health, Taipei, Taiwan

^8^Graduate Program in Biomedical Informatics, Yuan Ze University College of Informatics, Taiwan

^9^Department of Internal Medicine, National Taiwan University Hospital, Taiwan

**Correspondence:** Dr. Yen-Ling Chiu, Graduate Program in Biomedical Informatics, Yuan Ze University, Taiwan. E-mail: [yenling.chiu@saturn.yzu.edu.tw](mailto:yenling.chiu@saturn.yzu.edu.tw); Professor Wei-Shiung Yang, Graduate Institute of Clinical Medicine, College of Medicine, National Taiwan University, Taipei, Taiwan. E-mail: [wsyang@ntu.edu.tw](mailto:wsyang@ntu.edu.tw).

*: The authors contribute to this manuscript equally.

Key words: Leucine-rich α-2-glycoprotein 1, peripheral arterial disease, end-stage renal disease, peripheral arterial occlusive disease, cardiovascular disease

Number of figures: 2; Number of tables: 4; Number of supplementary figure:1; Number of references: 38; Abstract word count: 229; Total word count: 3,367.

**Supplementary Figure 1**

**Prevalence of cardiovascular comorbidities stratified by hsCRP level**

Percentage and standard error of patients with each specified co-morbidity among each hsCRP tertile group is shown. Comparisons were performed by chi-square test. *: p value < 0.05. NS: non-significant, p value > 0.05.
